# Cuproptosis-related genes score and its hub gene GCSH: A novel predictor for cholangiocarcinomas prognosis based on RNA seq and experimental analyses

**DOI:** 10.7150/jca.92327

**Published:** 2024-01-21

**Authors:** Rui He, Yihu Li, Pengcheng Jiao, Yingbin Huang, Shuyi Dong, Liqiu Mo, Xingyuan Jiao

**Affiliations:** 1 Organ Transplant Centre, the First Affiliated Hospital, Sun Yat-sen University, Guangzhou, Guangdong, China.; 2 Department of Pathology, Guanghua School of Stomatology, Sun Yat-sen University, Guangzhou, Guangdong, China.; 3 Department of Surgical Anesthesiology, the First Affiliated Hospital, Sun Yat-sen University, Guangzhou, Guangdong, China.

**Keywords:** Cholangiocarcinomas, Cuproptosis, Prognostic model, GCSH

## Abstract

**Background:** Recent researches have demonstrated that cuproptosis, a copper-dependent cell death mechanism, is related to tumorigenesis, progression, clinical prognosis, tumor microenvironment, and drug sensitivity. Nevertheless, the function and impact of cuproptosis in cholangiocarcinoma (CCA), remain elusive.

**Methods:** Utilizing data obtained from the Gene Expression Omnibus (GEO) and The Cancer Genome Atlas (TCGA-CHOL) datasets, we conducted subgroup typing of CCA according to cuproptosis-related genes (CRGs) and explored functional differences and prognostic value between groups. A CRG score was established considering clinical prognosis and gene expression. Furthermore, differences in the immune microenvironment, response to immunotherapy, metabolic patterns, and cancer progression characteristics between high- and low-risk groups were examined on the basis of these scores. In vitro experiments validated the function of the key gene glycine cleavage system protein H (GCSH) in cellular and tissues, respectively.

**Results:** Prognostic models established on the basis of subgroup genetic differences achieved satisfactory results in validation. Metabolic-related gene expression levels and tumor microenvironment distribution were significantly different between the high and low CRG groups. GCSH was revealed as the singular prognostic CRG in CCA (HR =6.04; 95% CI: 1.15-31.80). Moreover, inhibition of the cupcoptosis key gene GCSH attenuated the malignant ability of CCA cell lines in vitro, including cell proliferation, migration and invasion, and this function of GCSH may be achieved via JAK-STAT signaling in CCA.

**Conclusion:** The CRG scoring system accurately predicts prognosis and opens up new possibilities for cuproptosis-related therapy for CCA. The cuproptosis key gene GCSH has been preliminarily confirmed as a reliable therapeutic target or prognostic marker for CCA patients.

## Introduction

Cholangiocarcinoma (CCA) has emerged as an aggressive malignancy with escalating morbidity and mortality in recent years. Owing to the absence of typical early signs and specific screening/diagnostic indicators, most CCAs are diagnosed in the advanced terminal stages 1. Surgical resection currently stands as the sole curative option, yet merely 30% of patients become eligible for this procedure 2. Furthermore, the high postoperative recurrence rate (50% to 60% worldwide) translates into a less than 45% 5-year survival rate for CCA patients 3. Therefore, effective drug therapy assumes paramount importance for CCA patients who are ineligible for surgery or experience postoperative recurrence. Numerous studies underscore the close correlation between the genetic characteristics of CCA and its response to drug treatment 4. Consequently, genetic testing is now frequently employed to select suitable targeted and immunotherapeutic agents, aiming to bolster the efficacy of treatment when combined with chemotherapy and surgical resection 5. Despite the promising future of the anti-PD-1 monoclonal antibody pembrolizumab for end-stage CCA patients, its current clinical utilization is suboptimal, highlighting the ongoing need for more effective drugs against CCA 6.

Programmed cell death, encompassing apoptosis, pyroptosis, necrosis, and ferroptosis, has been a focal point in oncology research, offering new avenues for prognostic prediction and tumor treatment 7. A recent study in the journal Science revealed a new form of programmed cell death dependent on copper ions, termed "cuproptosis" 8. The underlying principle of cuproptosis mainly involves the tricarboxylic acid cycle, where copper ions can act directly on fatty acylated elements, leading to fatty acylated protein aggregation and the deletion of iron-sulfur cluster proteins. This ultimately culminates in cell death as a response to proteotoxic stress 9. With the presence of 4 enzymes, include dihydrolipoamide branched chain transacylase E2 (DBT), glycine cleavage system protein H (GCSH), dihydrolipoamide S-succinyltransferase (DLST) and dihydrolipoamide S-acetyltransferase (DLAT) 10, which control the entry of carbon into the tricarboxylic acid cycle, copper contributes to the lipoylation of mitochondrial proteins at the cellular level that is the key process resulting in ultimately cell death. Despite these insights, our understanding of cuproptosis in the pathogenesis, progression, and prognosis of CCA remains limited 11.

Furthermore, the role of cuproptosis in the tumor microenvironment (TME) should not be ignored, and thus the guiding value of cuproptosis in targeted therapy and immunotherapy warrants further exploration. There is mounting evidence supporting the extensive involvement of the TME in the genesis and progression of CCA, influencing the outcomes of radiotherapy, chemotherapy, targeted therapy, and immunotherapy 12. Individuals with the same tumor may exhibit various, complex TME phenotypes, resulting in disparate responses to drug therapy, including tumor-promoting and immune-evasive or tumor-suppressive and immune-enhancing phenotypes 13,14. In our study, we elucidated the role of CRGs in CCA through multi-omics analysis and established a predictive model for CCA patients. The model's reliability was further validated through prognostic analysis, gene mutation analysis, immune infiltration analysis, and drug sensitivity analysis between high and low-risk groups. Moreover, the key gene in cuproptosis, GCSH, has been preliminarily confirmed as a reliable therapeutic target or prognostic marker for CCA patients.

## Materials and Methods

### Data collection and processing

**Figure 1:**
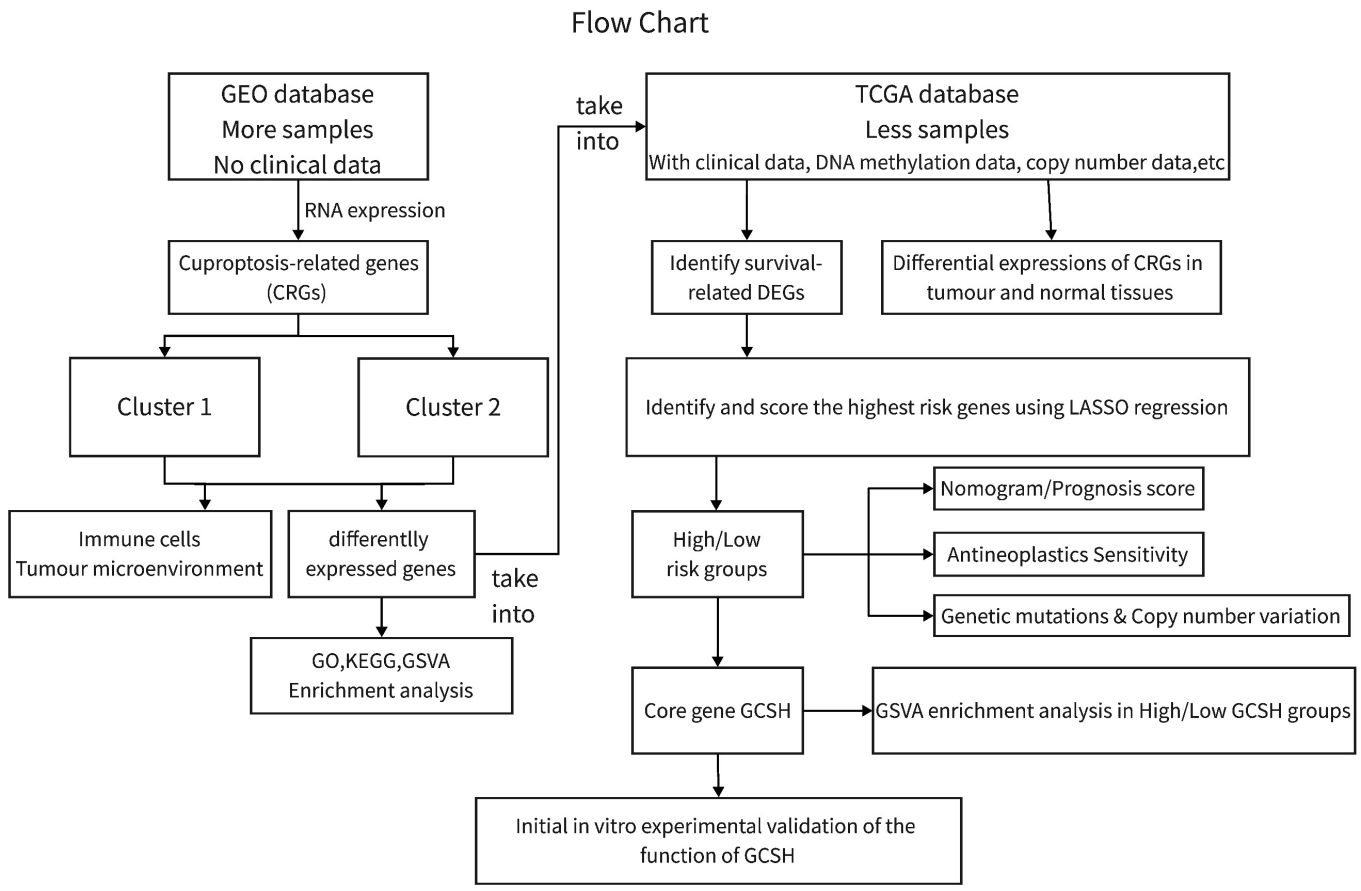
Flowchart for this study.

Genomic and clinical data for the CCA patients involved in this investigation were sourced from The Cancer Genome Atlas (TCGA) database (https://portal.gdc.cancer.gov/, accessed on October 15, 2022) and the Gene Expression Omnibus (GEO) database (https://www.ncbi.nlm.nih.gov/gds/, accessed on October 15, 2022). We conducted analyses on the GEO CCA cohort GSE26566 (n=104) and the TCGA-CHOL cohort (n=35) using R (version 4.2.2) and the R Bioconductor software package. Gene expression data from the database were uniformly converted to transcripts per million kilobases (TPM) values prior to subsequent exploration. The study process is depicted in Figure 1. As this study involved the mining of public databases, formal permission and informed consent from the ethical review board were not required.

### Identifying distinct subpopulations of cuproptosis in CCA

Thirteen CRGs were selected following the criteria outlined by Tsvetkov et al 8. Subsequently, unsupervised consensus clustering analysis was conducted utilizing the R package "Consensus Clusterplus" to categorize CCA patients into distinct subgroups depending on CRGs expression levels. The Cumulative Distribution Function (CDF) curve exhibited a stable rise during clustering, successfully grouping all isoforms into an adequate number of samples for subsequent analysis.

### Different expressed genes (DEGs) analysis and functional enrichment analysis between cuproptosis subgroups

DEGs with fold changes greater than 2 and adjusted p-values less than 0.05 between distinct cuproptosis subgroups were selected utilizing "GEO2R" (https://www.ncbi.nlm.nih.gov/geo/info/geo2r.html). Subsequently, Gene Ontology (GO), Kyoto Encyclopedia of Genes and Genomes (KEGG) pathways, and Gene Set Variation Analysis (GSVA) were conducted to elucidate the biological functions and molecular mechanisms underlying the diversity among cuproptosis subtypes.

### Analysis of differences in TME between subgroups of cuproptosis

The proportion of 22 immune cells in the TME between different cuproptosis subtypes was calculated by CIBERSORT. Box plots are drawn using the "limma" and "ggpubr" packages.

### Construction of prognostic model for CCA patients

Incorporating DEGs into the TCGA-CHOL cohort, we employed univariate Cox regression to discern the effect of DEGs expression on overall survival (OS) time. Utilizing the identified DEGs, a CRG prognostic model was established by LASSO Cox regression analysis. Subsequently, CRG risk scores were calculated for all patients based on the prognostic model to include them into high-risk and low-risk groups based on the median risk scores, and their OS times were compared using Kaplan-Meier analysis. Finally, a principal component analysis (PCA) was conducted on DEGs employing the "prcomp" function within the "stats" package.

### Analysis of differences in clinical characteristics between risk subgroups of cuproptosis

Chi-square test and Wilcoxon rank-sum test were utilized to analyze the differences in clinical information, such as age, gender, and TNM stage, between high and low risk groups related to cuproptosis.

### Construction and verification of predictive nomogram

A predictive nomogram was constructed using the "rms" package. Receiver operating characteristic (ROC) curves were utilized to evaluate the reliability of the nomogram. Calibration plots were generated to illustrate differences between predicted survival events and actual results.

### Mutation and copy number variation (CNV) analysis between subpopulations

The "maftools" package was used to analyse somatic mutations in CCA. CNVs in CCA were analyzed using the "copynumber" package.

### Methylation alterations drug susceptibility analysis

Methylation analysis was performed using the SMART database (http://www.bioinfo-zs.com/smartapp/). The semi-inhibitory concentration (IC50) of common chemotherapeutic agents against CCA was calculated using the “pRRophetic” package.

### Cell lines

The human cholangiocarcinoma cell line HCCC-9810 (CL-0095) was obtained from EK-Bioscience (Shanghai, China) and the human cholangiocarcinoma cell line RBE (CL-0191) were obtained from Procell Life Science & Technology Co., Ltd. (Wuhan, China). All cells were cultured in RPMI 1640 medium (Gibco) with 10% FBS and 1% penicillin-streptomycin in a 37℃, 5% CO2 incubator.

### Human samples

The human tumor samples used in this study were acquired from 116 patients underwent CCA resection at the First Affiliated Hospital, Sun Yat-sen University, from 2015 to 2022. All samples were used only for experimental purposes. All processes involving human samples were permitted by the Clinical Research and Experimental Animal Ethics Committee of the First Affiliated Hospital of Sun Yat-sen University. Clinical information of these patients was provided in Supplementary Table 1.

### In vitro overexpression and knockdown of GCSH

For in vitro overexpression of GCSH in CCA cells, the custom-made pCMV6-AC-GFP vector containing human GCSH cDNAs was purchased from Origene (#RG217886; Wuxi, China). 3 unique 27mer GCSH-siRNAs were also purchased from Origene (#SR301770; Wuxi, China) and were used according the manufacture's protocol. The overexpression or knockdown efficiency was verified by western blotting with a specific antibody.

### Western blot and antibody

After the standard extraction of total cellular proteins, lysates were quantified and subjected to electrophoresis on SDS-PAGE gels. Subsequently, they were transferred to PVDF membranes (Millipore) and immunoblotted with primary antibodies overnight at 4°C. Following this, PVDF membranes were incubated with secondary antibodies for 2 hours at room temperature. Antibody signals were detected using an enhanced chemiluminescence system. The antibodies and their respective sources were as follows: GCSH (Servicebio, #GB114523, 1:1000), JAK1 (CST, #3344, 1:1000), p-JAK1 (CST, #74129, 1:1000), STAT3 (CST, #9139S, 1:1000), p-STAT3 (CST, #9145S, 1:1000), GAPDH (Proteintech, #66009-1-lg, 1:1000).

### CCK8 Assay

Cell proliferation activity was assessed using the CCK-8 Detection Kit (CellCook, #CT01C) following the manufacturer's guidelines. Cells were plated in 96-well microplates at a density of 1 × 10^4^ cells per well in 100 μL of RPMI-1640 medium supplemented with 10% FBS. Subsequently, 10 μL of CCK-8 reagent was introduced into each well and incubated for 2 hours. Six replicates were employed for each experimental group. Absorbance at 450 nm was measured using a multifunctional microplate detector (Infinite F200 Pro), with cell-free wells serving as blanks.

### Wound-Healing Assay

CCA cells in the logarithmic growth stage were resuspended in RPMI-1640 medium containing 10% FBS, reaching a concentration of 5×10^5^/mL. 30 μL of cell suspension was dispensed into the scratch chambers on each side in 6-well plate respectively. Following cell monolayer adherence, the scratch chamber was meticulously removed with tweezers and washed with PBS to eliminate any suspended cells. Next, 2 mL of serum-free RPMI-1640 medium per well was added, and the cells were cultured in a 37°C, 5% CO2 incubator. Microscopic images were captured at 0 and 48 hours, with each experiment being conducted thrice.

### Transwell Assay

CCA cells were suspended in serum-free RPMI-1640 medium at a concentration of 10^5^/mL. Subsequently, 600 μL of RPMI-1640 medium containing 10% FBS was introduced into the lower chamber, and 200 μL of the cell suspension was placed in the upper chamber. The upper chamber was carefully immersed into the lower chamber liquid using sterile forceps. Following a 24-hour incubation at 37°C in an incubator, the upper chamber was withdrawn and rinsed three times with an appropriate volume of PBS. Cells in the upper compartment were then stained with crystal violet, visualized under an electron microscope, and photographed. Each experiment was replicated three times.

### Immunohistochemistry (IHC)

After epitope retrieval, H2O2 treatment, and nonspecific antigen blocking, deparaffinized and dehydrated tissues were incubated with rabbit anti-human monoclonal GCSH (Servicebio, #GB114523, 1:500) or rabbit anti-human monoclonal p-STAT3 (CST, #9145S, 1:300) overnight at 4°C. Next, tissues were incubated with secondary antibody. After that, using DAB staining kit (Servicebio, #GB1212) detected the signals. Mean gray values of cytoplasmic staining were extracted by ImageJ (1.54d) software for further linear regression analysis.

### Statistical analysis

Using cox regression models evaluated the independent prognostic value of the predictive model. All statistical analyses of common databases were conducted using R version 4.2.2. Experimental data analyses for this study were performed using GraphPad Prism 8.0. Unless otherwise stated, the data were presented as the mean ± s.e.m. Statistical differences between two groups were identified by two-tailed Student's t-test. p<0.05 was considered statistically significant. P values were showed as: *, p<0.05; **, p<0.01; ***, p<0.001; ****, p<0.0001.

## Results

### Differential expression and DNA methylation of CRGs in CCA

**Figure 2:**
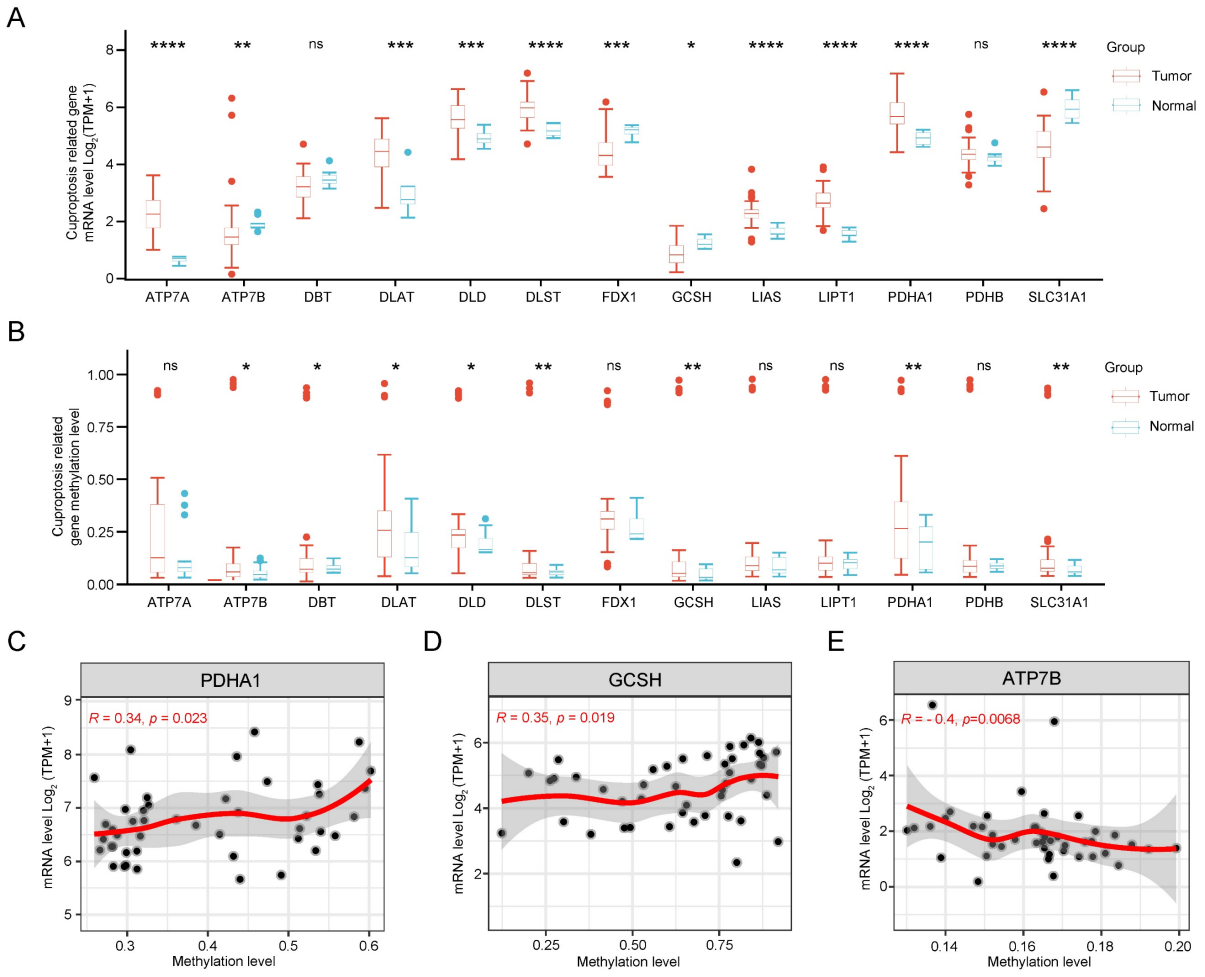
Gene expression and DNA methylation levels of CRGs in CCA. (A) Expression levels of 13 CRGs in CCA compared with normal tissues. (B) DNA methylation status of 13 CRGs in CCA compared to normal tissues. Correlation analysis of gene expression level and methylation level of PDHA1 (C), GCSH (D), ATP7B (E) in CCA.

Initially, we scrutinized the expression patterns of CRGs in CCA. As illustrated in Figure 2A, the expression levels of SLC31A1, FDX1, DBT, GCSH, and ATP7B were down-regulated, while PDHA1, LIPT1, DLD, DLST, LIAS, DLAT, and ATP7A showed up-regulation compared to normal tissue. Subsequently, significant differences in DNA methylation stages of GCSH, DBT, DLAT, ATP7B, DLD, DLST, PDHA1, and SLC31A1 between tumor and normal tissue were observed (Figure 2B). Notably, the expression levels of PDHA1, GCSH, and ATP7B exhibited a clear correlation with their methylation status (Figure 2C). Finally, methylation sites, particularly cg09787394 and cg17746819, were identified as prognostically relevant in CCA (Supplementary Table 2). Therefore, dysregulation and methylation status of CRGs could be critical in the pathogenesis of CCA.

### Identification of CRGs clusters in CCA

**Figure 3:**
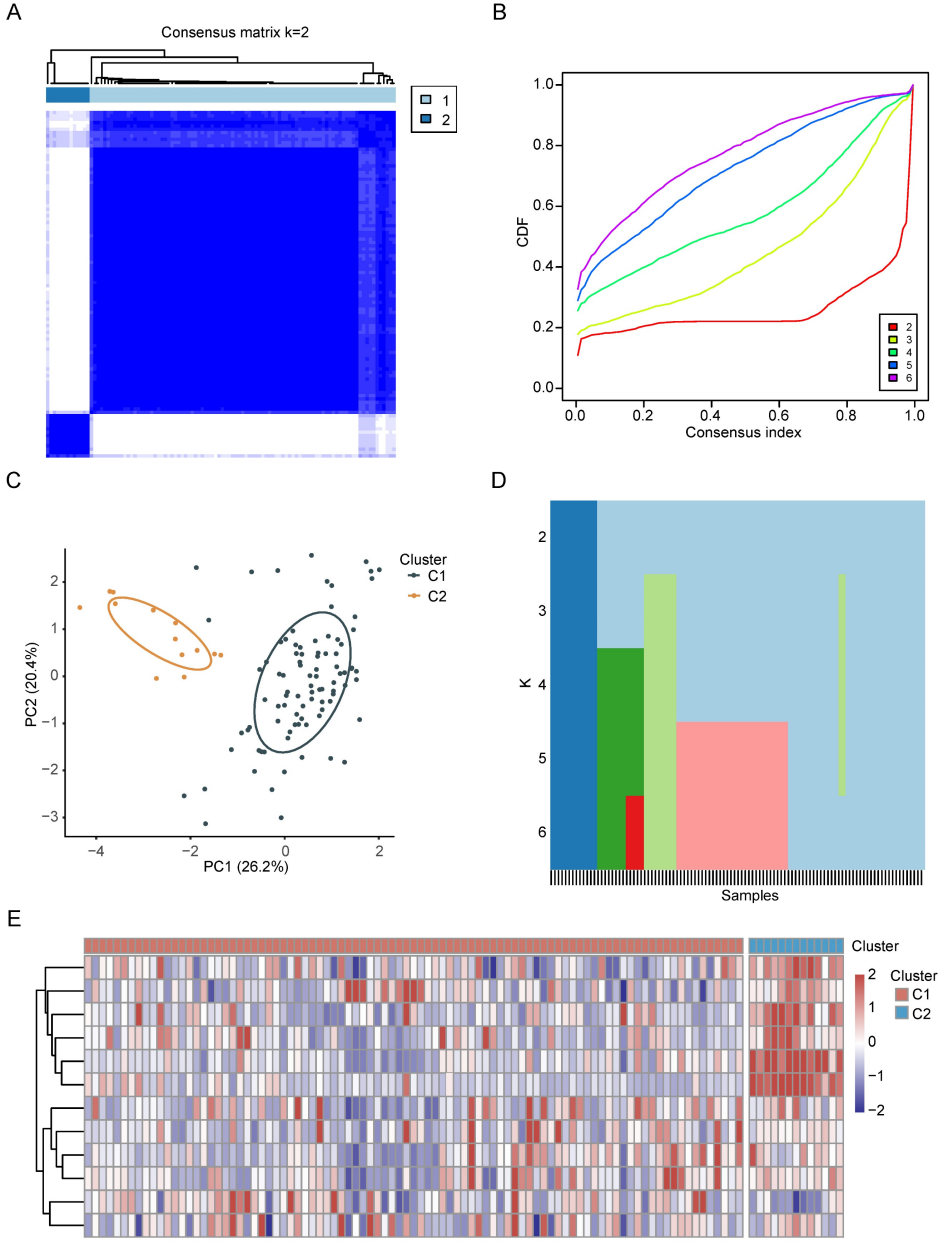
The characterization of CRGs in CCA. (A-C) The consensus matrix heatmap of two clusters (k=2) and their respective regions. (D) PCA analysis illustrating the two subtypes based on transcriptional expression in CCA patients. (E) Unsupervised clustering of CRGs in CCA cohorts.

Based on the preceding findings, CRGs emerge as pivotal factors in the context of CCA. Consequently, we performed an intensive study on the characterization of CRGs in CCA. First, we categorized CCA patients employing the consensus clustering algorithm based on RNA expression patterns of CRGs. The optimal value of k, crucial for clustering efficacy and genetic correlation, was determined through a comprehensive analysis across values ranging from 2 to 6 (see Figure 3A, Supplementary Figure 1A-D). Notably, k = 2 yielded the most optimal clustering outcomes (Figure 3B), leading to the designation of two subgroups as C1 (n = 91) and C2 (n = 13) (Figure 3C). As depicted in the heatmap, PCA further confirmed the presence of two distinct subgroups (Figure 3D). The expression patterns of CRGs in these two subgroups are visually represented in Figure 3E.

### Association of CRGs signature with metabolic characteristics in CCA

**Figure 4:**
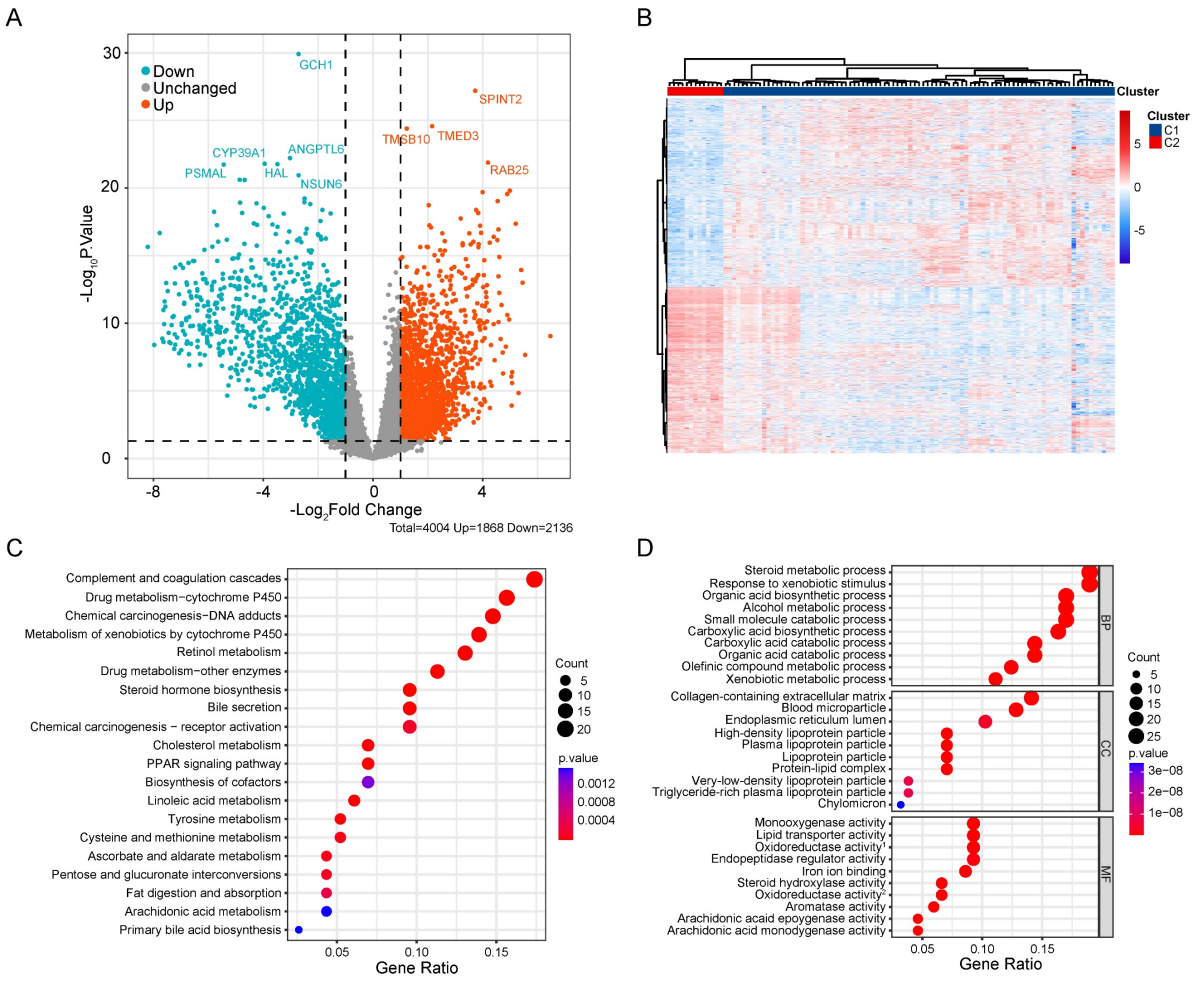
Comprehensive analysis of DEGs in CCA. (A) Volcano map of DEGs in CCA cuproptosis subgroups. (B) Differential expression of genes in two cuproptosis clusters**.** GO (C) and KEGG (D) enrichment analysis of DEGs between two subtypes of CCA.

**Figure 5:**
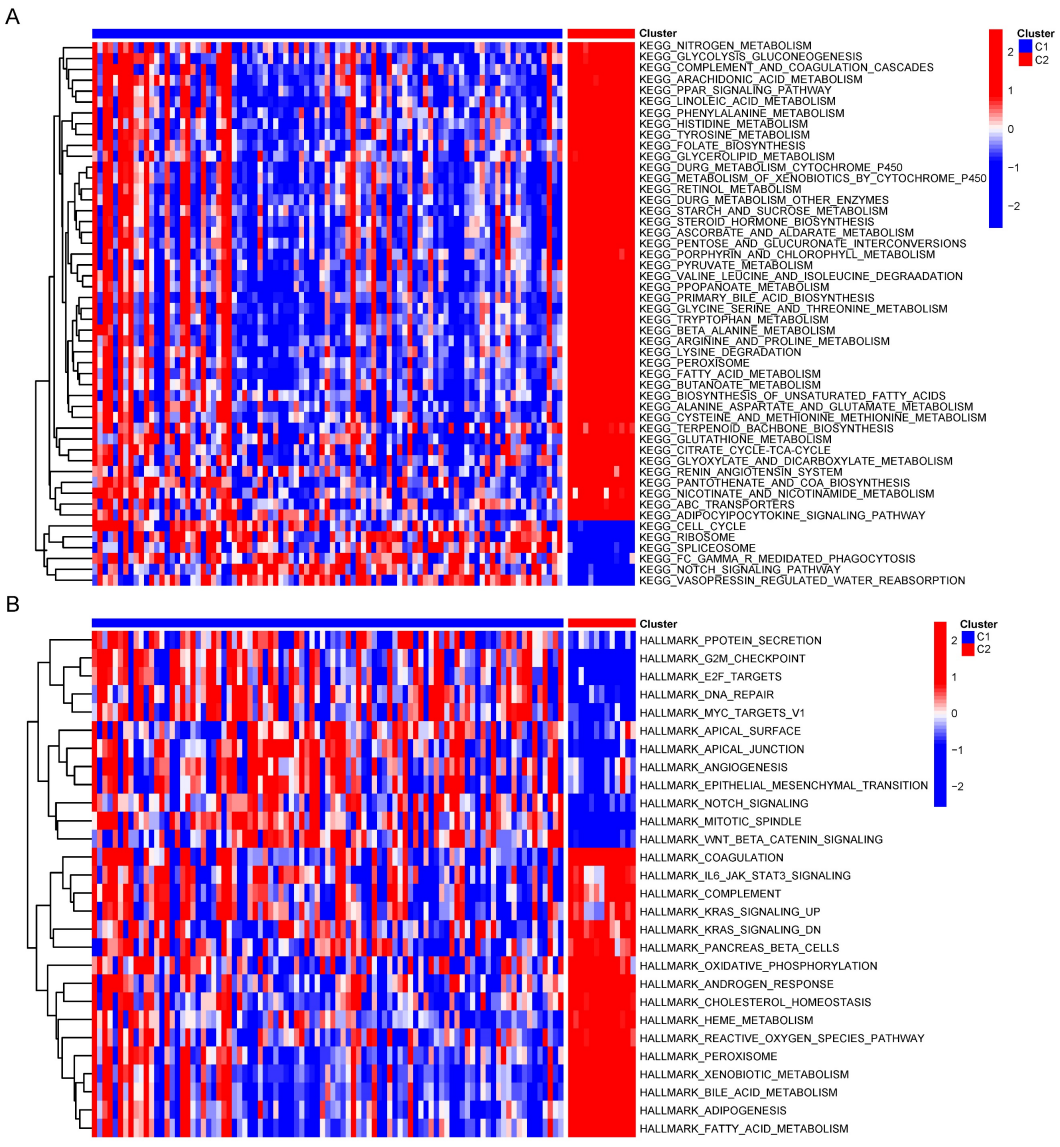
GSVA of DEGs between two clusters.

To compare the biological behavior and functional differences between these two cuproptosis subgroups, DEGs screening and further functional enrichment analysis was performed. A total of 4004 DEGs, encompassing 1868 upregulated genes and 2136 downregulated genes, emerged between the two groups (Figure 4A,B). Notably, 172 genes, exhibiting a fold change in expression greater than 5 and a significance level of p < 0.001, were singled out for Gene Ontology (GO) and Kyoto Encyclopedia of Genes and Genomes (KEGG) enrichment analyses. The GO analysis highlighted the involvement of most DEGs in the regulation of the tumor metabolic microenvironment (Figure 4C). Similarly, KEGG analysis unveiled associations between the majority of DEGs and various metabolic and oncogenic pathways (Figure 4D). Furthermore, GSVA using GSE26566 data confirmed distinct characteristics between the two cuproptosis groups. The C1 group exhibited marked enrichment in cell cycle, mitosis, angiogenesis, and epithelial-mesenchymal transition, while the C2 group displayed higher enrichment in the metabolism of multiple nutrients, inflammation-related signaling pathways, and certain established oncogenes (Figure 5A,B). These findings underscore the potential disparities in cell metabolism behavior and the cell cycle between the two cuproptosis groups, prompting further analysis of these DEGs in the TCGA-CHOL database.

### CRGs were potential immunotherapeutic target for CCA

**Figure 6:**
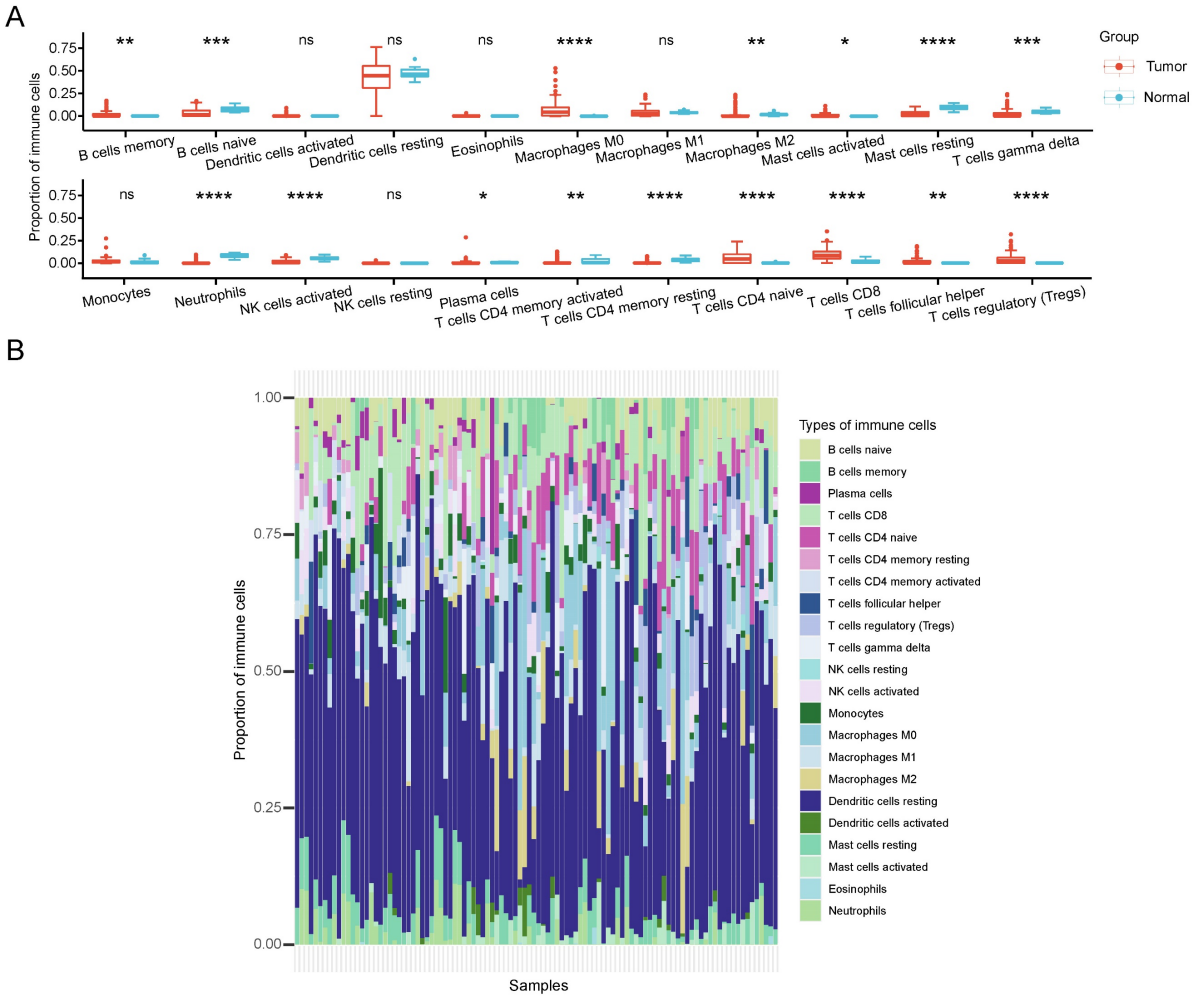
TME characteristics between cuproptosis subtypes in CCA. (A) The quantitative analysis of various immune cell in the TME of 2 cuproptosis clusters. (B) Immune cell composition of every sample in CCA cohorts.

Immunological therapy has become increasingly indispensable in the treatment of CCA patients recently. To elucidate the role of CRGs in the CCA TME, the proportion differences of 22 immune cells between different cuproptosis subtypes was calculated by CIBERSORT. Our analysis revealed that the two clusters differed significantly in the proportion of various immune cells infiltrated (Figure 6A). In comparison with the C2 group, the C1 group exhibited significantly higher infiltration levels of B cells memory, macrophages M0, mast cells activated, plasma cells, T cells CD4 naive, T cells CD8, T cells follicular helper, and T cells regulatory (Tregs). Conversely, B cells naive, Macrophages M2, Mast cells resting, T cells gamma delta, neutrophils, NK cells activated, T cells CD4 memory activated, and T cells CD4 memory resting showed markedly lower infiltration levels in C1 compared to C2. Figure 6B represents the proportion of immune cells in each sample. Based on these findings, we hypothesize that the C2 subtype may induce a more robust immune response by stimulating copper-induced cell death, resulting in an increased presentation of antigens.

### High CRGs risk score indicated the poor prognosis of CCA patients

**Figure 7:**
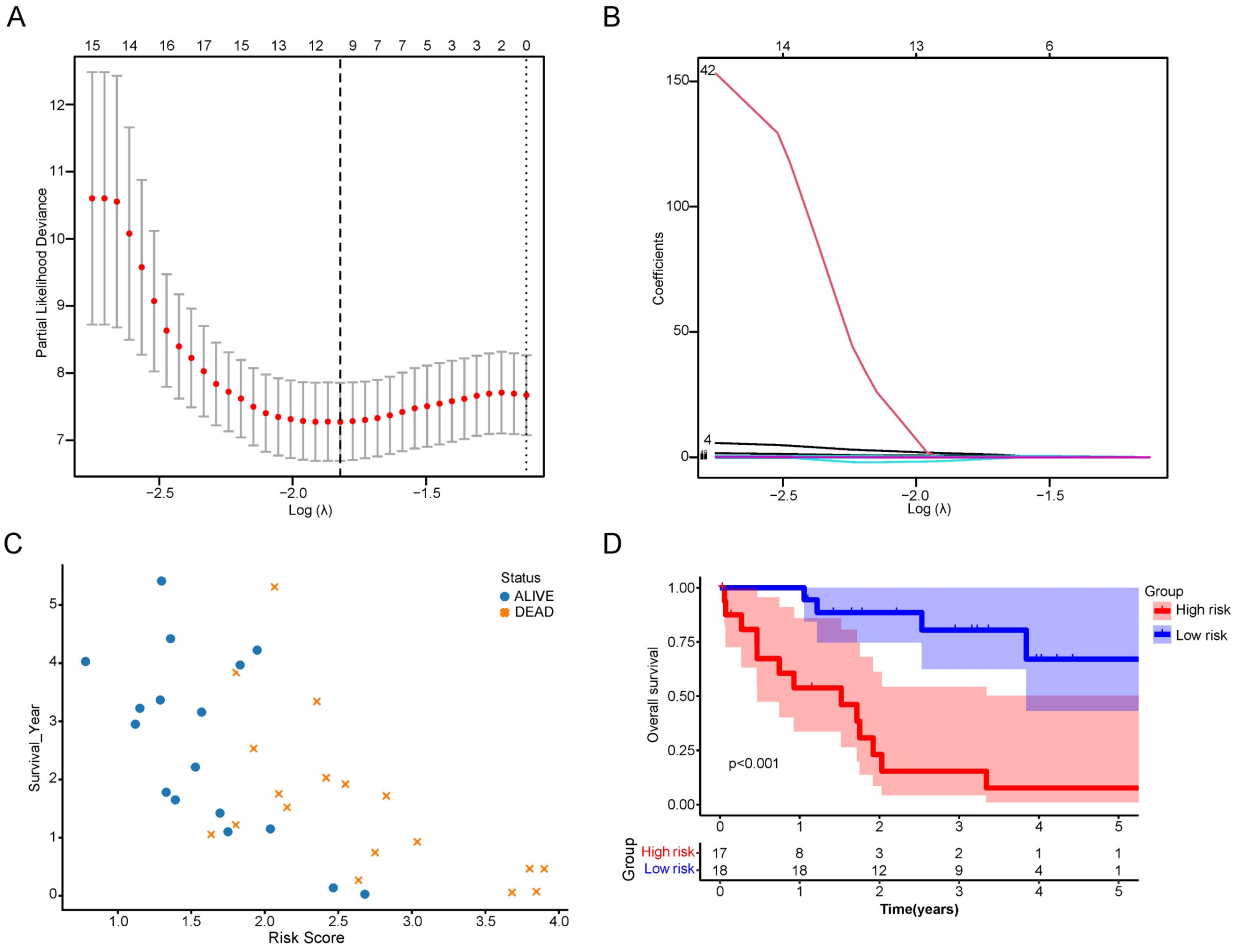
Construction of a scoring system for cuproptosis. (A,B) LASSO regression identified the best risk genes. (C) Scatterplot of patient prognosis in relation to CRS. (D) Survival curve of CCA patients in different risk groups.

The DEGs between the two CRGs clusters were utilized to formulate a cuproptosis scoring model. Employing LASSO regression with an optimal λ value, 68 risk genes were identified and selected (Figure 7A, B). Subsequently, a Multivariate Cox regression analysis based on Akaike information criterion (AIC) values was conducted, yielding 10 key genes. Among these, seven were identified as risk genes (EFCAB1, CDADC1, WNK4, WNT10A, FGFBP2, AGTR1, and LBX2), while three were recognized as protective genes (RNASEH2A, HAMP, and ELAC1). The Cuproptosis Risk Score (CRS) for CCA based on these 10 genes was calculated using the formula: CRS = Exp(EFCAB1) × (1.43309) + Exp(CDADC1) × (0.63383) + Exp(RNASEH2A) × (-1.14489) + Exp(HAMP) × (-0.00037) + Exp(WNK4) × (0.52956) + Exp(WNT10A) × (0.00610) + Exp(FGFBP2) × (0.17787) + Exp(ELAC1) × (-0.14684) + Exp(AGTR1) × (0.04918) + Exp(LBX2) × (0.28862). Following CRS calculation, patients were categorized into high- and low-risk groups depending on the median CRS. It was observed that with increasing CRS values, the number of deceased patients significantly rose (Figure 7C). Survival analysis further indicated that the high-risk group had significantly lower survival times and survival rates than the low-risk group (Figure 7D).

### Nomogram involved in clinicopathological features and CRG risk score can effectively predict OS of CCA patients

**Figure 8:**
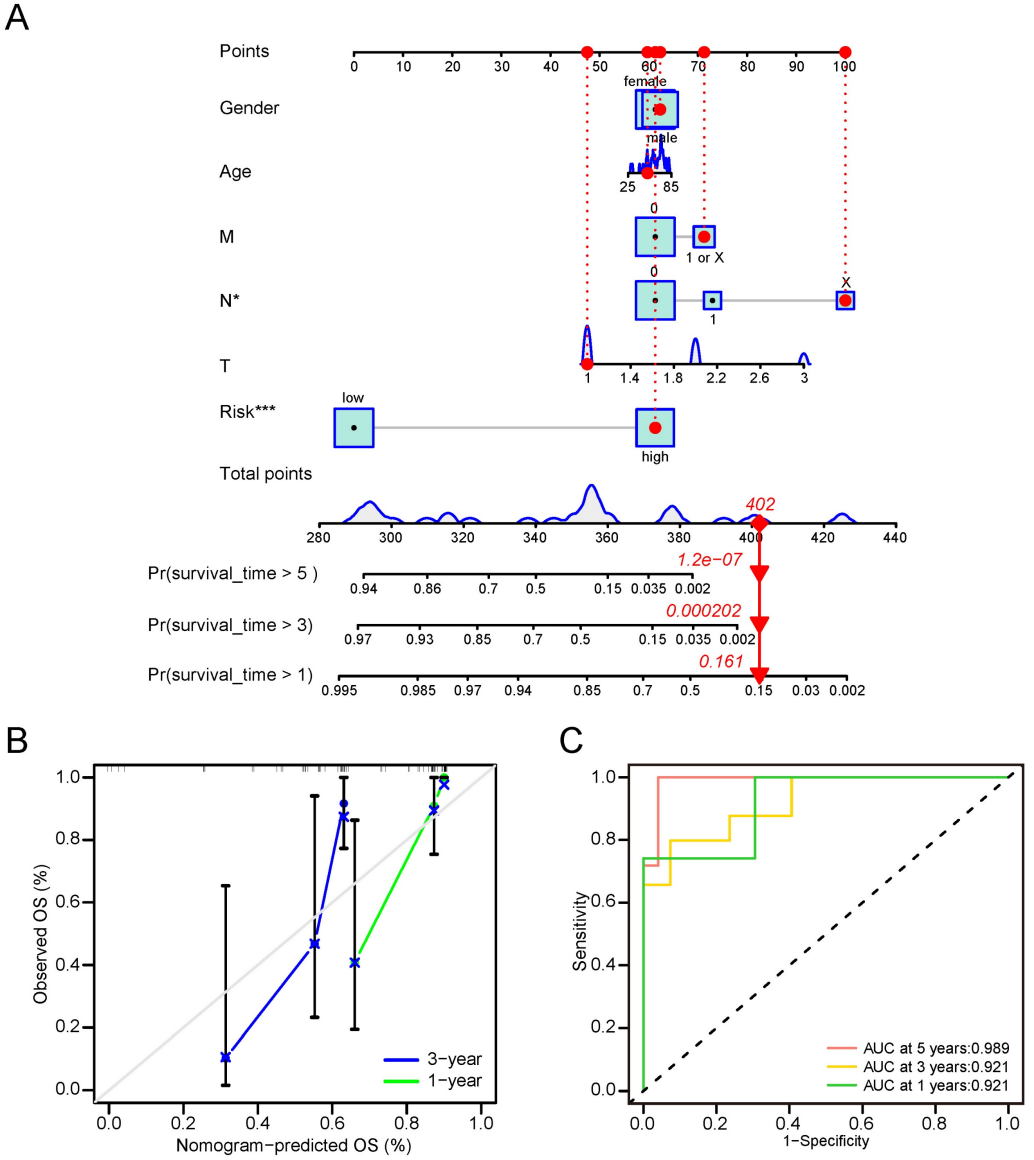
Nomogram involved in clinicopathological features and CRG risk score for CCA. (A) 1-, 3-, and 5-years OS in CCA patients predicted by nomogram. (B) 1- and 3-years OS of CCA patients predicted by calibration curves. (C) 1-, 3-, and 5-years OS of CCA patients predicted by ROC curves.

To formulate a robust model for predicting the OS of CCA patients, we developed a predictive nomogram that incorporated both clinical-pathological features and CRG risk scores. The analysis demonstrated that the N stage and CRS were independent risk factors for OS in CCA (Figure 8A). Calibration curves showed that our nomogram reasonably predicted 1- and 3-year OS in CCA patients, although the accuracy of 5-year OS assessment was limited due to the small number of patients living beyond 5 years (Figure 8B). The AUC results reflected satisfactory accuracy for 1-, 3- and 5-year OS, with AUC values of 0.921, 0.921, and 0.989, respectively (Figure 8C). These findings underscored significant differences in clinical outcomes between high-risk and low-risk groups. Notably, the CRS proved to stratify CCA OS more effectively than the TNM stage.

### Mutation, CNV and drug susceptibility analysis of two subtypes

**Figure 9:**
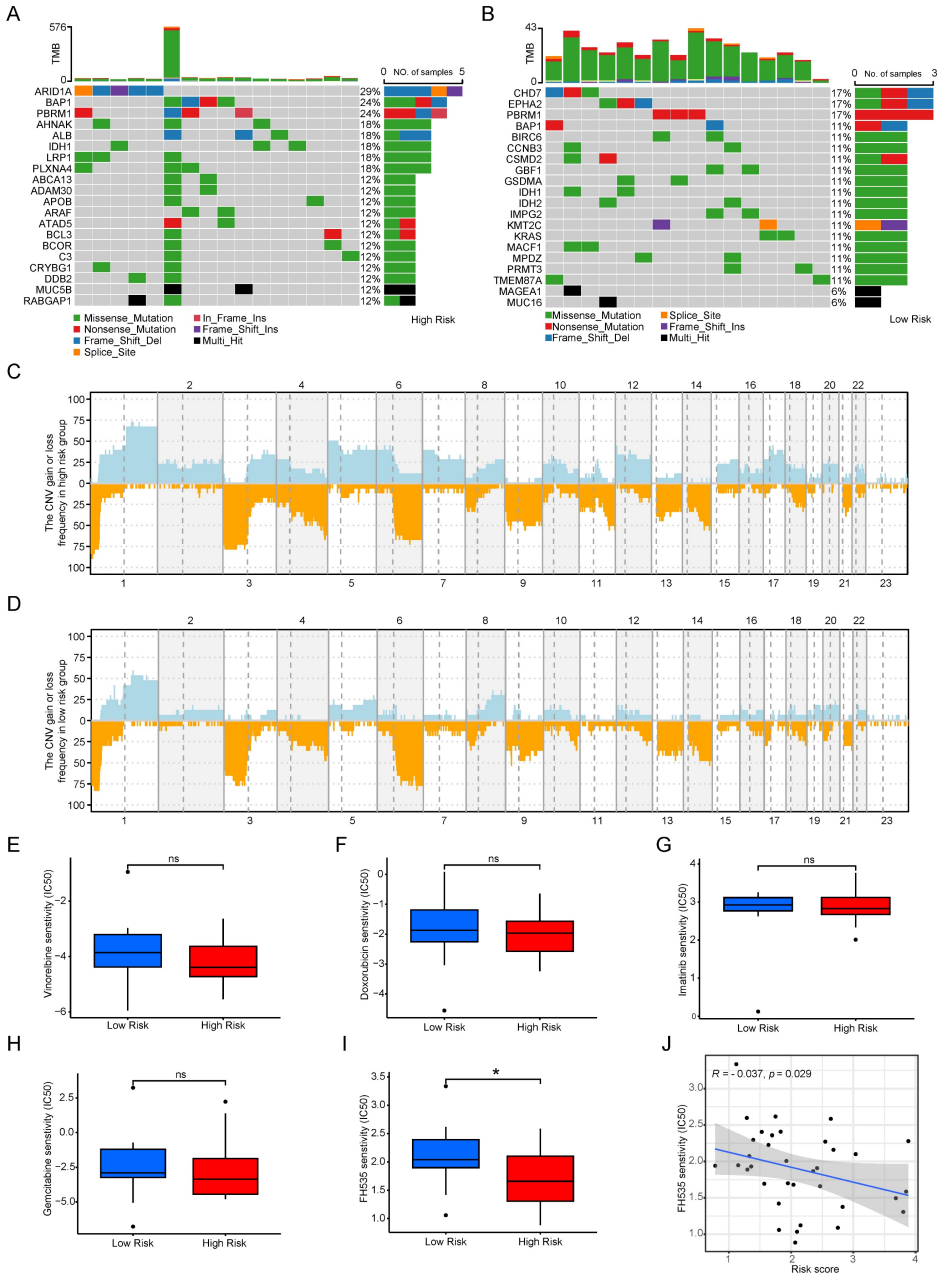
Mutation, CNV and drug susceptibility analysis of two subtypes. (A, B) Waterfall plots showing somatic mutation signatures in the copper apoptosis risk model. (C,D) Variant frequencies of genes in 2 risk groups. The frequency of changes is represented by the height of the column. Above the horizontal line indicates profit, and below the horizontal line indicates loss. (E-J) The representative examples of cuproptosis risk model assisting antitumor drug candidate selection.

The microenvironment's impact on drug treatment aside, genomic mutations play a pivotal role in determining drug effectiveness. Consequently, we scrutinized distinctions in tumor somatic mutations, CNV, and immunotherapy responses among various cuproptosis score groups. Initially, we characterized gene mutations in the two groups and subsequently calculated the tumor mutation burden (TMB). Notably, the TMB was higher in the high-risk group than in the low-risk score group, and the top 20 genes with the highest mutation frequencies exhibited considerable variability between the groups (see Figure 9 A, B). Figures 9C and 9D depict the CNV of all genes in Cholangiocarcinoma (CCA), revealing that CNV loss and gain frequencies were more prevalent and consistent in the high-risk group. Moreover, predictive analyses of drugs targeting CCA and their susceptibility in subgroups were conducted. No significant differences were observed between the two groups regarding sensitivity to commonly used clinical drugs such as gemcitabine, doxorubicin, imatinib, and vinorelbine (see Figures 9E-H), except for FH535 (see Figures 9i, J). These results underscore the utility of the risk model constructed based on Cuproptosis Risk Score (CRS) in predicting drug responses in patients with CCA.

### GCSH as the main CRGs enhances the malignancy of CCA

**Figure 10:**
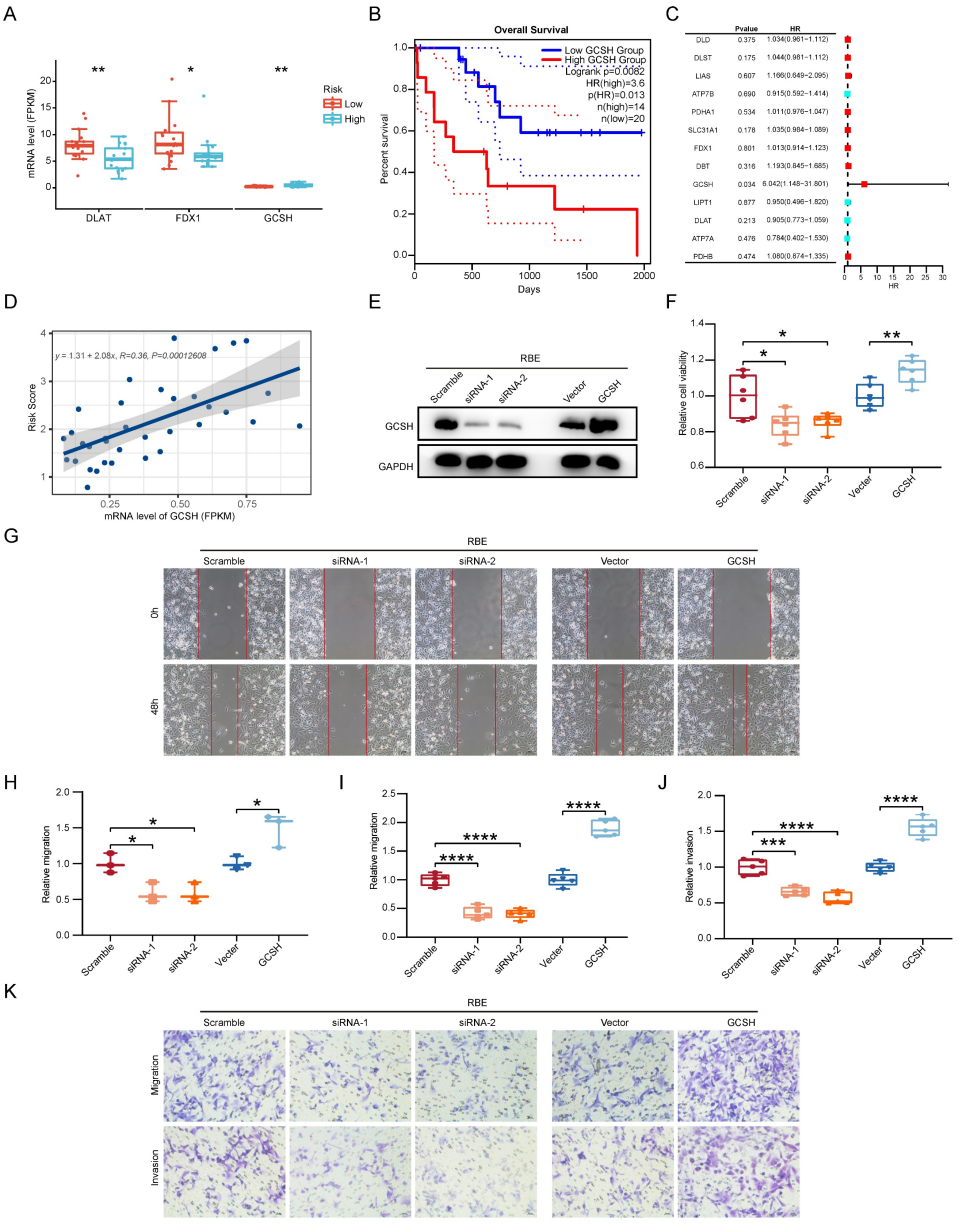
GCSH as the main CRGs enhances the malignancy of CCA. (A) The protein expression comparison of FDX1, DLAT and GCSH between the two subgroups of CCA samples. (B) Correlation analysis of gene expression level and OS of FDX1, DLAT and GCSH in CCA. (C) Random forest analysis of CRGs in CCA. (D) Linear regression analysis of GCSH between GCSH expression levels and risk scores. (E) Representative western blots showing the GCSH protein levels in RBE cells. (F) The cell proliferation capacity of RBE cells using CCK8 assay. (G and H) The cell migratory capacity of RBE cells using Wound Healing assay. (I-K) The cell migratory and invasive capacity of RBE cells using transwell assay.

Given the pivotal role of CCA, we directed our focus toward identifying the CRG with the most substantial impact on clinical outcomes. Through a reassessment of 13 CRGs within the two subgroups of CCA samples, we observed statistically significant differences in the expression levels of FDX1, DLAT, and GCSH (p < 0.05) (Figure 10A). Specifically, only GCSH exhibited a notable association with Overall Survival (OS) in CCA patients (Figure 10B). Subsequently, we gauged the significance of these genes in clinical prognosis. In contrast to prior investigations 11, GCSH, rather than FDX1, assumed a central role in the cuproptosis signature, as indicated by random forest analysis (Figure 10C). Linear regression analysis further revealed a noteworthy linear positive trend between GCSH expression levels and risk scores (Figure 10D). Increased GCSH expression corresponded with higher risk scores, signifying a poor prognosis and identifying GCSH as a risk factor for CCA. Furthermore, in vitro perturbation experiments were conducted to validate the functional role of GCSH in CCA. Utilizing the CCK8 assay, siRNA-mediated knockdown of GCSH (Figure 10E, Supplementary Fig. 2A) apparently suppressed the growth of RBE and HCCC-9810 cells (p < 0.05), while GCSH overexpression (Figure 10E, Supplementary Fig. 2A) yielded the opposite result (p < 0.01) (Figure 10F, Supplementary Fig. 2B). Wound healing assays (p < 0.05) (Figure 10G, H, Supplementary Fig. 2C, D) and transwell assays (p < 0.001) (Figure 10I-K, Supplementary Fig. 2E-G) further demonstrated that GCSH inhibition markedly reduced the migration and invasion capacity of CCA cells. Conversely, GCSH overexpression significantly heightened the malignancy of RBE and HCCC-9810 cells. These findings underscore that knocking down of GCSH expression effectively suppresses the proliferation, migration, and invasion of CCA cells. Consequently, silencing GCSH expression to disrupt copper-induced death presents an intriguing avenue for eradicating tumors.

### Cuprotosis GCSH in CCA could enhance tumor malignancy through JAK-STAT signaling

**Figure 11:**
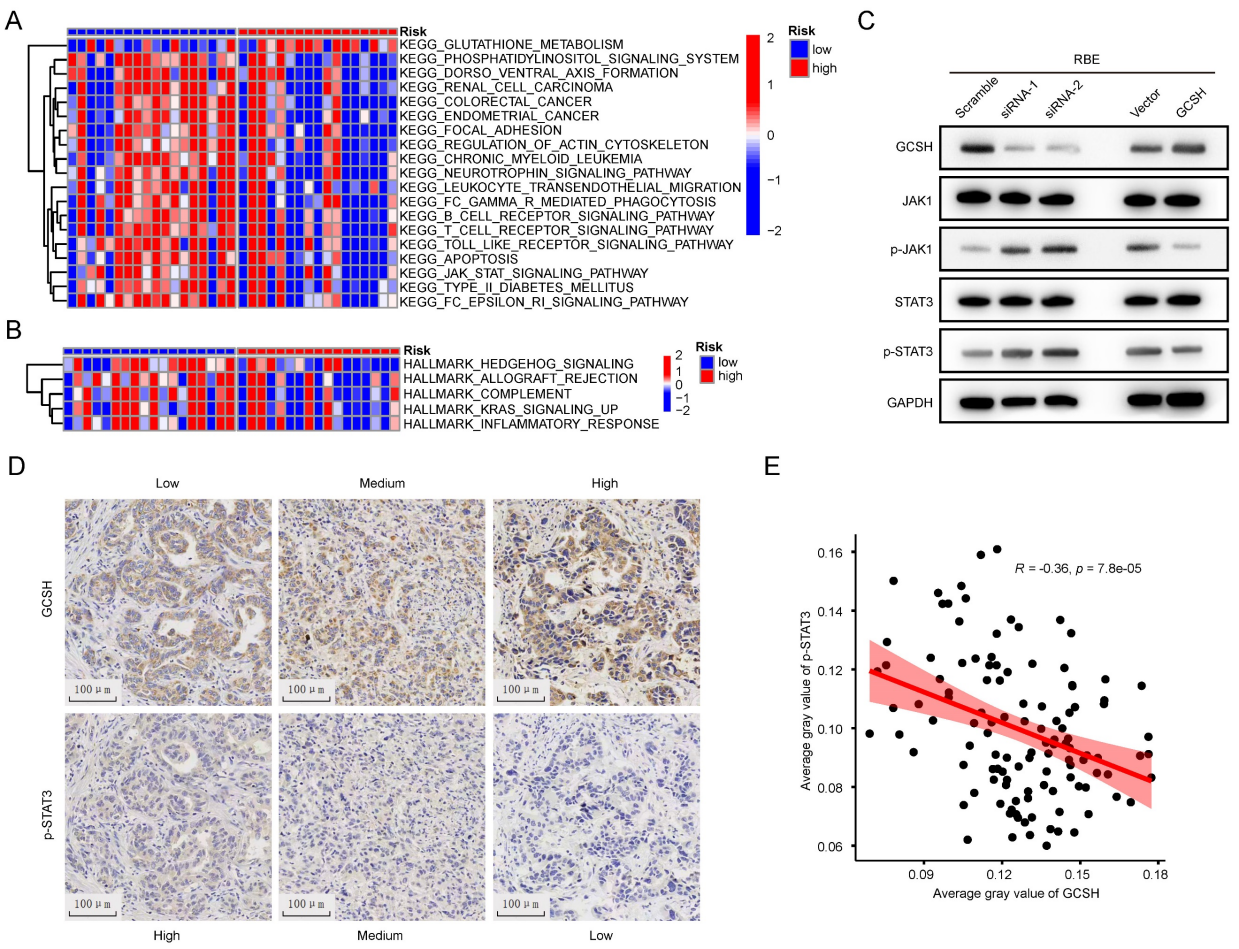
Cuprotosis in CCA could enhance tumor malignancy though JAK-STAT signaling. (A,B) GSVA of DEGs in in low and high GCSH groups. (C) Representative western blots showing JAK-STAT signaling related proteins expression level in RBE cells. (D) Representative IHC images of GCSH and p-STAT3 expression in tumor tissues of CCA patients. (E) Correlation analysis between GCSH and p-STAT3 protein expression level.

To elucidate the potential mechanism of GCSH expression in the prognosis of CCA, GSVA was conducted to compare the GCSH^Low^ and GCSH^High^ groups. Hallmark pathway enrichment analysis revealed significant suppression of the inflammatory response, complement, and K-ras signaling pathways in the GCSH^High^ group (Figure 11A). KEGG pathway enrichment analysis further indicated inhibition of the JAK-STAT signaling pathway, B cell receptor signaling pathway, T cell receptor signaling pathway, Toll-like receptor signaling pathway, and Fc-γ-R-mediated phagocytosis in the GCSH^High^ group (Figure 11B).

Combining the outcomes of these two analytical approaches, we hypothesize that GCSH potentially augments tumor malignancy through the JAK-STAT signaling pathway in CCA. This aligns with existing reports that highlight the role of the JAK-STAT signaling pathway in mediating inflammatory responses and its confirmed involvement in tumor development 15,16,17,18. Indeed, we observed that GCSH knockdown activated the JAK-STAT signaling pathway in RBE (Figure 11C) and HCCC-9810 cells (Supplementary Figure 2H). Conversely, upregulation of GCSH expression significantly suppressed the JAK-STAT signaling pathway (Figure 11C, Supplementary Figure 2H). Additionally, we identified a negative correlation between GCSH expression and p-STAT3 in the tumor tissues of CCA patients using IHC (Figure 11D). Linear regression analysis further substantiated a remarkable and negative correlation between GCSH and p-STAT3 expression (R=-0.36, p < 0.0001) (Figure 11E). These findings suggest that GCSH can suppress the activation of the JAK-STAT signaling pathway, thereby promoting CCA progression.

## Discussion

Cuproptosis emerges as a newly identified programmed cell death pathway distinct from traditional modes such as apoptosis, pyroptosis, and ferroptosis 19. Interestingly, both copper chelators and copper ionophores have undergone clinical trials as antitumor agents 20, 21. Copper's critical role in tumor cell proliferation, angiogenesis, and cancer metastasis has been reported 22. Subsequent to the discovery of cuproptosis, mounting evidence suggests its significant involvement in various tumors, inflammation, and immune responses 23. While numerous studies confirm the link between abnormal copper accumulation and CCA, the current research lacks a systematic molecular mechanism, with few providing detailed analysis of cuproptosis in CCA progression. Therefore, characterizing tumor subpopulations based on multiple Copper-Related Genes (CRGs) derived from cuproptosis principles may aid in identifying potential prognostic features and influencing immune cell infiltration, guiding immunotherapeutic strategies for CCA. Our study's results reveal altered methylation and transcriptional regulation levels of most CRGs in CCA tissues compared to normal tissues. Based on these findings, we hypothesize that cuproptosis plays a significant role in CCA progression. We classified 104 CCA samples into two subgroups (CRG groups C1 and C2) based on 13 CRGs in the GEO database. Notably, the immune infiltration characteristics and tumor signaling pathways significantly differed between the two subtypes.

Group C1 exhibited marked enrichment in cell cycle, mitosis, angiogenesis, and epithelial-mesenchymal transition, while group C2 displayed heightened activity in the metabolism of multiple nutrients, inflammation-related signaling pathways, and established oncogenes. These results indicate potential differences in cell metabolism and the cell cycle between the two cuproptosis groups, and this evidence highlights the role of CRGs in cancer metabolic reprogramming 24,25,26.

Simultaneously, we observed significant disparities in immune cell infiltration between cuproptosis subgroups. Group C1 showed increased enrichment of CD8 T cells, B cells memory, macrophages M0, activated mast cells, plasma cells, T cells CD4 naive, T cells follicular helper, and Tregs, while group C2 exhibited enrichment in B cells naive, macrophages M2, resting mast cells, T cells gamma delta, neutrophils, activated NK cells, T cells CD4 memory activated, and T cells CD4 memory resting. Given that current research considers CD8 T cells activated by neoantigens as main effector cells, playing a tumor-killing role and representing a hotspot for anti-tumor immunotherapy 27, the immune microenvironment between the two clusters appears different. Nevertheless, how cuproptosis affects functional and metabolic changes in antitumor immune cells remains unclear.

Considering the substantial discrepancies between the two clusters and recognizing the importance of CRGs, we included Differentially Expressed Genes (DEGs) in the TCGA-CHOL database for further analysis. Utilizing cuproptosis-related differential genes, we established a robust CRG prognostic score, demonstrating its predictive power. Stratifying CCA patients into high and low-risk groups using this prognostic score revealed significant differences in prognoses. These findings underscore the cuproptosis score as an excellent indicator for survival prognosis in CCA patients and a potential guide for clinical treatment.

Subsequent analysis uncovered significant variations in genetic variant levels between the high and low-risk groups. Patients with higher cuproptosis scores exhibited higher Tumor Mutational Burden (TMB) levels and a poorer prognosis, indicating that the cuproptosis score independently predicts immunotherapy response efficacy. Building on this, we explored the difference in antitumor drug susceptibility between the two groups. Only FH535 showed significant differences in sensitivity between groups, while commonly used CCA chemotherapy drugs, including gemcitabine 28, cisplatin 29, oxaliplatin 30, fluorouracil 31, and capecitabine 32, showed no significant distinctions. These results suggest that the cuproptosis risk model effectively guides the medication regimen for CCA patients.

Furthermore, our research identified GCSH as the sole prognostic Copper-Related Gene (CRG) in CCA. Low GCSH expression impeded malignant abilities in CCA cells in vitro, including migration, invasion, and proliferation. Gene Set Variation Analysis (GSVA) revealed that high GCSH levels suppressed the inflammatory response and JAK-STAT signaling pathway. In vitro experiments confirmed that GCSH knockdown activated the JAK-STAT signaling pathway, while GCSH overexpression produced the opposite effect. Given the known impact of JAK-STAT signaling on tumor survival, proliferation, and invasion, this pathway has been considered as a therapeutic target in various cancers, such as hematological cancer 33, breast cancer 34, prostate cancer 35, and brain cancer 36. Additionally, other researchers highlighted STAT3 as a critical regulator in CCA, influencing angiogenesis, immunosuppression, and tumor invasion 37,38. Our findings suggest that GCSH enhances CCA malignancy by inhibiting the JAK-STAT pathway, thus reactivating the copper death pattern. While GCSH presents a promising target for CCA therapy, the intricate details of this relationship require further investigation.

However, it is important to acknowledge that this study has limitations. All analyses were performed retrospectively using data from public databases, available pathological tissues, and in vitro experiments. Therefore, additional in vivo experimental research is essential to further support our findings.

## Conclusion

The CRGs scoring system proves accurate in predicting prognosis and introduces novel prospects for cuproptosis-related therapy in Cholangiocarcinoma (CCA). Our study, uniquely in the context of CCA, unveils the prognostic significance of CRGs, establishing correlations with CCA development, immune infiltration, and drug sensitivity. Notably, the key cuproptosis gene, GCSH, has been preliminarily confirmed as a reliable therapeutic target and prognostic marker for CCA patients.

## Supplementary Material

Supplementary figures and tables.
