# Supplementary material for: Cuproptosis-related genes score and its hub gene GCSH: A novel predictor for cholangiocarcinomas prognosis based on RNA seq and experimental analyses
Source: J Cancer. 2024 Jan 21;15(6):1551–67. doi: 10.7150/jca.92327 (PMC10869970; doi:10.7150/jca.92327)
Supplement: Supplementary file 1 — Supplementary figures and tables. [file jcav15p1551s1.pdf]

Supplementary Figure

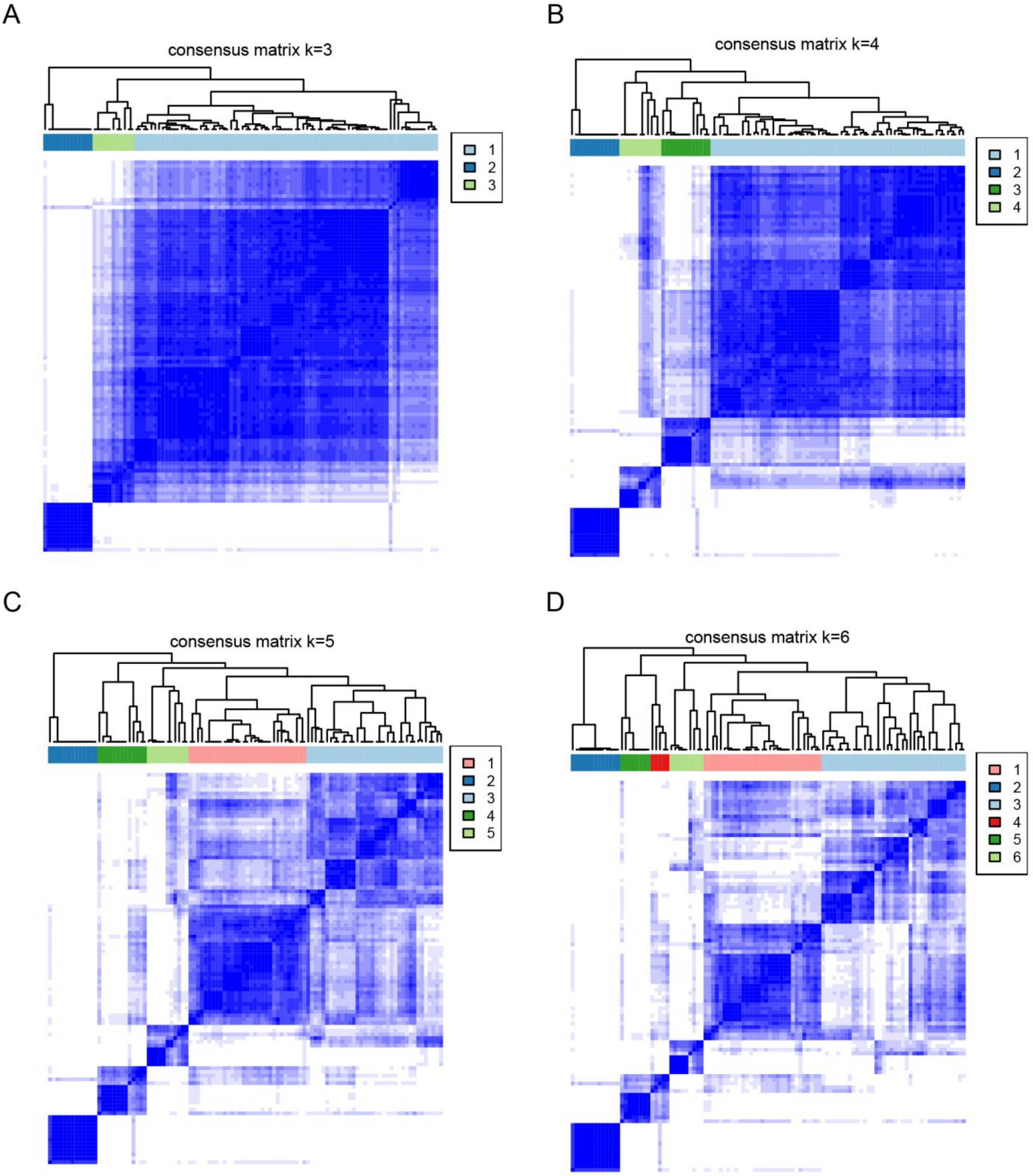

Supplementary Figure1 The clustering effect of different k values in CCA. (A) k=3. (B) k=4. (C) k=5. (D) k=6.

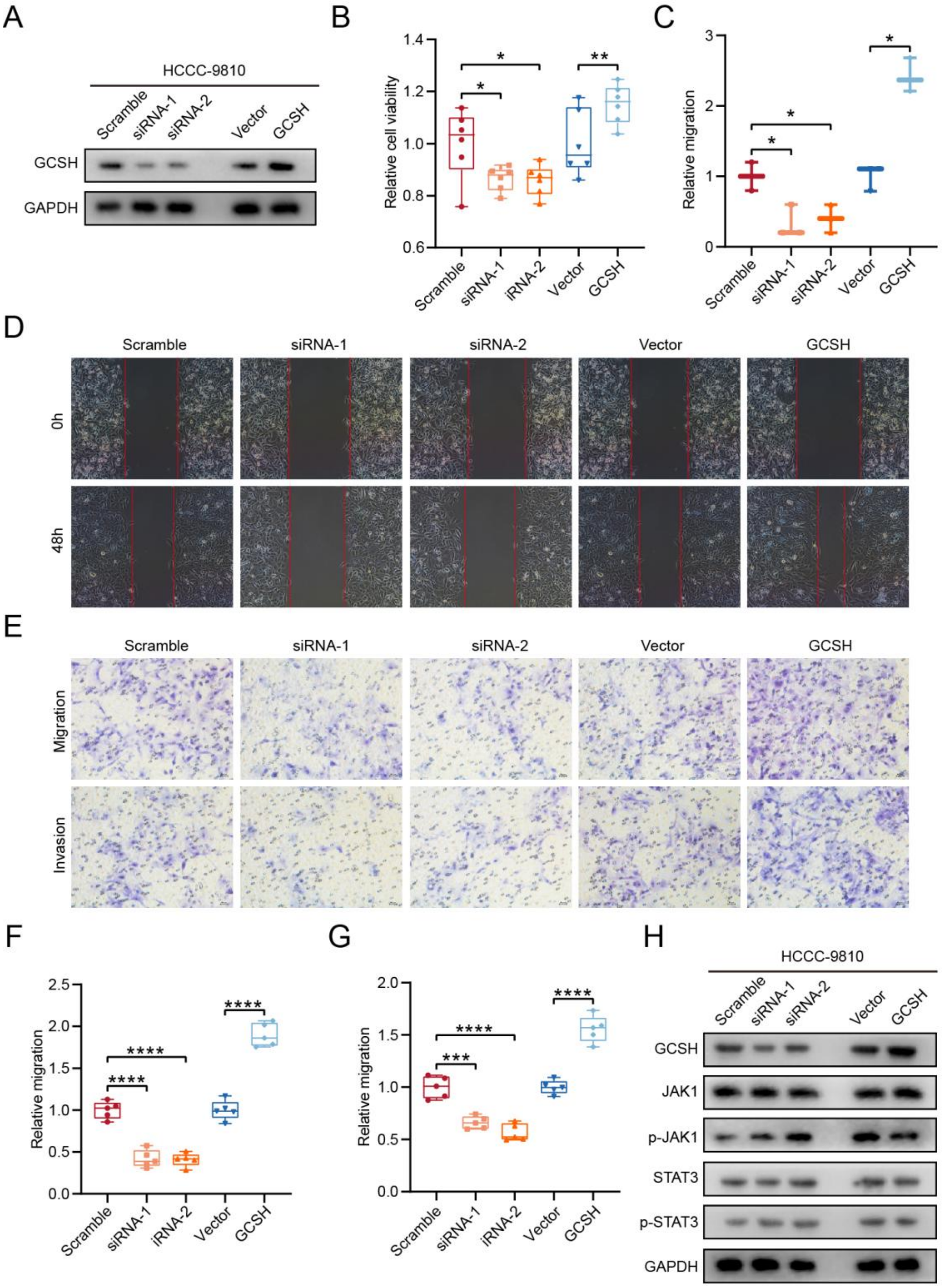

Supplementary Figure2 GCSH functional verification in HCCC-9810 cells. (A) Representative western blots showing the GCSH protein levels in HCCC-9810 cells. (B) The cell proliferation capacity of HCCC-9810 cells using CCK8 assay. (C and D) The cell migratory capacity of HCCC-9810 cells using Wound Healing assay. (E-G) The cell migratory and invasive capacity of HCCC-9810 cells using transwell assay. (H) Representative western blots showing JAK-STAT signaling related proteins expression level in HCCC-9810 cells.

Supplementary Table

Supplementary Table 1.The clinical characteristic of 116 patients with CCA

Variable		Mean ± s.e.m/Number(%)
Age (years)		60±12.05
Gender		
	Male	68 (58.6%)
	Female	48 (41.4%)
Tumor number		
	Single	86 (74.1%)
	Multiple	30 (25.9%)
Tumor size (cm)		
	≤5	59 (50.9%)
	＞5	57 (49.1%)
Differentiation grade		
	High	0
	Medium	64 (55.2%)
	Medium-Low	43 (37.1%)
	Low	9 (7.7%)
MVI		
	No	81 (69.8%)
	Yes	35 (30.2%)
Lymphatic metastasis		
	No	75 (64.7%)
	Yes	41 (35.3%)
Distant metastasis		
	No	110 (94.8%)
	Yes	6 (5.2%)
Stage		
	Ⅰ	50 (43.1%)
	Ⅱ	19 (16.4%)
	Ⅲ	43 (37.1%)
	Ⅳ	4 (3.4%)

Supplementary Table 2. Two methylation sites associated with prognosis in CCA

ID	HR	HR.95L	HR.95H	pvalue
cg09787394	4.70E-11	4.97E-21	0.4448724	0.0424506
cg17746819	1811.0535	1.2934668	2535754.8	0.0423989
